# On the Injection Molding Processing Parameters of HDPE-TiO_2_ Nanocomposites

**DOI:** 10.3390/ma10010085

**Published:** 2017-01-20

**Authors:** Abdel-Hamid I. Mourad, Mohammad Sayem Mozumder, Anusha Mairpady, Hifsa Pervez, Uma Maheshwara Kannuri

**Affiliations:** 1 Mechanical Engineering Department, College of Engineering, UAE University, Al Ain 15551, UAE; ahmourad@uaeu.ac.ae; 2 Chemical & Petroleum Engineering Department, College of Engineering, UAE University, Al Ain 15551, UAE; mairpadyanu@uaeu.ac.ae (A.M.); hifsa.p@uaeu.ac.ae (H.P.); umamahesh422@uaeu.ac.ae (U.M.K.)

**Keywords:** HDPE/TiO_2_ nanocomposites, injection molding parameters, structural changes, mechanical properties, thermal stability

## Abstract

In recent years, the development and use of polymeric nanocomposites in creating advanced materials has expanded exponentially. A substantial amount of research has been done in order to design polymeric nanocomposites in a safe and efficient manner. In the present study, the impact of processing parameters, such as, barrel temperature, and residence time on the mechanical and thermal properties of high density polyethylene (HDPE)-TiO_2_ nanocomposites were investigated. Additionally, scanning electron microscopy and X-ray diffraction spectroscopy were used to analyze the dispersion, location, and phase morphology of TiO_2_ on the HDPE matrix. Mechanical tests revealed that tensile strength of the fabricated HDPE-TiO_2_ nanocomposites ranged between 22.53 and 26.30 MPa, while the Young’s modulus showed a consistent increase as the barrel temperature increased from 150 °C to 300 °C. Moreover, the thermal stability decreased as the barrel temperature increased.

## 1. Introduction

Polymers have been used as starting materials for several different applications, and polymeric composites have formed a particularly interesting niche in biomedical engineering. Currently there are a variety of metallic and ceramic materials that are being pursued as micro or nanofillers in a polymer matrix [1]. Incorporation of additives into the polymeric matrix are mostly used to enhance the existing properties, however, it can also be considered a cost effective method for value added products [1,2]. New hybrid polymeric materials are being continually developed by the addition of fillers that fine tune the physical, chemical, and mechanical properties of the composites [1,3]. The effect of different nanofillers can be observed from the improved properties of the polymeric nanocomposites, however, fine tuning the process parameters of injection molding itself is also another important factor that must be considered.

Polyethylene has been widely studied in combination with metallic nanoparticles for their combined advantage in hard tissue replacement and tissue engineering [3,4]. Specifically, high density polyethylene (HDPE), a thermoplastic material, has shown great promise as a matrix component of bone implants; its structure consists of long chains of carbon and hydrogen atoms bonded together with variable branching throughout which determines its mechanical properties [5]. Given the versatility of HDPE, many research groups have studied the effect of incorporation of fillers into the polymer matrix, including titanium dioxide, aluminum oxide, hydroxyapatite, and zirconia etc. that resulted in biocomposites with improved physical properties and enhanced cellular responses in bone-like cells [3,6,7,8,9,10].

Metallic fillers such as titanium, zinc, and copper are known to improve the thermal and electrical conductivities of polymer matrices [1,11]. Among them, titanium has been used as implant material since the 20th century, and its alloys have many uses in orthopedic and dental research [9]. The popularity of titanium is attributed to its excellent biocompatibility, corrosion resistance, and high strength; nevertheless, its high modulus of elasticity limits its use as sole material for implants and hence the incorporation of pure titanium or titanium dioxide in polymers has resulted in feasible polymeric composites [9]. A high modulus causes bone resorption, and therefore, minimizing this effect results in successful integration of implants in the body [3,9]. Other types of fillers such as quasi-crystalline (QC), hydroxyapatite, polyimidazole fibers, and aluminum oxide have also been added to HDPE matrix to fabricate specialized composites [1,3,12,13].

Nanocomposites based on polymeric matrix are fabricated by various techniques including injection molding [3,14], compression molding [15,16], in situ polymerization [17,18], sol-gel [19], and sintering [9]. Among them, in situ polymerization involves the dispersion of inorganic nanoparticles in a monomer phase as a first step, followed by bulk phase polymerization [20]. The resulting solution produced polymeric nanocomposites with well-dispersed nanoparticles and good flowing properties [17]. However, a limitation of this technique shows that the mixture consists of unstable nanocomposites that may revert into a different morphology other than the expected [20]. Moreover, this process is not very common in industry and is mainly used for thermosetting polymers. In addition, compression molding is another technique used for fabricating nanocomposites; it involves a mold cavity in which the polymer blend is poured and results in composites of various dimensions. The processing conditions include very high pressure and the fabricated nanocomposites may not always have a uniform consistency [16]. Furthermore, most compression molding techniques require pre-treatment of the nanoparticles with curing.

Injection molding offers higher production cycles than compression molding thermoplastics [21]. This technique has become one of the most significant industrialized techniques in the field of polymer composites processing. The process provides high production rates, repeatable high tolerances, and low labor cost, and is anticipated to hold greater potential in the fabrication of uniform, good quality polymer micro- and sub-micrometer structures [22]. Injection molding can be used in a variety of applications in both commercial and research fields. A large number of polymer parts with complex geometry and good dimensional accuracy can be automatically manufactured by injection molding [23].

Liu et al. fabricated Ti-HDPE composites based on a sintering method that was carried out between a temperature of 1000–3000 °C and time ranging between 1 h and 12 h [9]. Sintering, powder compaction and sol-gel are all alternative techniques to produce polymeric composites, however, the operating conditions (temperature, pressure etc.) and time needed are far in excess of those of injection molding [24]. As a polymer matrix, high density polyethylene (HDPE) is well-known for its semi-crystalline structure which is closely bound to the overall macroscopic properties of the polymer such as mechanical and thermal characteristics [25]. In injection molding, HDPE undergoes an isothermal crystallization process which is a complex phenomenon occurring due to the varying shear and temperature gradients that occur while the polymer melts and fills the mold [23]. The various gradients contribute to the formation of different layers with different levels of crystallinity that affect the mechanical properties of the composites. In addition, the residence time (i.e., the time duration that the polymer spends in the barrel) along with barrel temperature, and the crystallization process of the polymer, especially HDPE, become affected [23]. In a study by Bociaga and Palutkiewicz [24], it was found that mold temperature had the greatest impact on the mechanical properties and surface structure. They investigated the effect of different injection molding conditions and concluded that low injection temperature, or barrel temperature, resulted in polymeric molded parts that consisted of a solid skin layer and a fine cellular core. Also, low weight HDPE parts were obtained at low injection temperature due to the formation of numerous tiny pores and the resultant high viscosity of the melting plastic, since temperatures used were close to the melting point of HDPE [26]. In his study, Mourad [27] concluded that the modulus of elasticity, yield strength, and tensile toughness decreased with increasing polyethylene (PE) content in the PE/PP blends of different concentrations and different process parameters. In a similar study by Mourad et al. [28] on the thermal characteristics of thermally treated and untreated VLDPE and isotactic PP blends (iPP), it was found through TGA and DSC analysis that addition of VLDPE resulted in a decrease in the melting temperature, heat of fusion, and percent crystallinity of the iPP based blends.

Since injection molding is a commonly used technique for mass production in industrial applications, it is imperative to study the different operating conditions so that the process is optimized [29]. Therefore, the aim of the current study was to prepare HDPE-TiO_2_ nanocomposites with a 5% constant nanofiller concentrations through injection molding. The two main parameters studied were the barrel temperature and the residence time fixed at 250 °C and 50 min respectively when either of the parameter was being studied. The effects of these process parameters were investigated based on the measured mechanical properties (i.e., Young’s modulus, tensile strength, and percent elongation) and thermal properties (e.g., crystallization and degradation temperatures) of the produced composites. Moreover, structural analyses such as X-ray Diffraction (XRD) and Fourier Transform Infrared (FTIR) spectroscopy were also used as tools to differentiate any structural changes of the polymer matrix. The surface morphology and titanium dioxide distribution was studied by means of scanning electron microscopy (SEM) along with energy dispersive spectroscopy (EDS) mapping of different elements along the surface of the injection molded substrates.

## 2. Materials and Methods

Commercially available high-density polyethylene (HDPE) and titanium dioxide (TiO_2_) were supplied by Sigma-Aldrich (Munich, Germany). The supplied HDPE has a transition temperature (softening point) of 123 °C and melt flow index of 2.2 g/10 min and the TiO_2_ nanoparticles (average particle size ~150 nm) of puriss grade have a melting temperature of >350 °C.

### 2.1. Synthesis of HDPE-TiO_2_ Nanocomposites

The HDPE nanocomposites having 5% TiO_2_ were subjected to high shear mixing to ensure even dispersion of TiO_2_ in the polymer matrix. The homogenous HDPE/TiO_2_ mixture was then transferred into the hopper of a gas assisted injection molding machine. After waiting for a specific amount of time, the sample was injected into the mold at constant air pressure and the mold temperature was maintained at 100 °C. The extruded pellets were injected and molded into dumbbell shaped nanocomposites. In the present paper, the effects of two operating conditions were analyzed. When studying the effect of barrel temperature (150 °C, 175 °C, 200 °C, 250 °C, and 300 °C) on the nanocomposites properties, residence time was kept constant at 50 min. Similarly, while evaluating the influence of residence time (30, 40, 50, 60, and 70 min) on properties of nanocomposites, the barrel temperature was maintained at 250 °C.

### 2.2. Characterization

#### 2.2.1. Mechanical Testing

Injection molded dumbbell shaped specimens were evaluated for their tensile properties using a 5 kN load cell universal testing machine. A nominal gauge length of 20 mm was used, extended up to fracture with a cross head speed of 5 mm/min to obtain stress-strain curves. From the tensile curve, the tensile strength, elastic moduli, and elongation to break were determined.

#### 2.2.2. Thermal Analyses (TGA and DSC)

To illustrate the thermal stability of the fabricated nanocomposites, thermogravimetric analysis (TGA) and differential scanning calorimetry (DSC) were carried out on the fabricated HDPE-TiO_2_ nanocomposites. One composite sample of each operating condition (i.e., barrel temperature and residence time) was considered for the thermal analyses, and its melting point, crystallization point, percentage of crystallization, and degradation temperature were determined.

TGA was carried out in a TA instrument (Model Q 50, TA Instruments, New Castle, DE, USA) to quantitatively evaluate the weight change observed for 10 mg of the sample as a function of temperature and time. The temperature profile programmed in TGA consisted of heating range 25 °C to 600 °C. The degradation temperature was varied from room temperature to 600 °C under the influence of nitrogen and the heating rate was maintained in the system at 10 °C·min^−1^.

The crystallization and melting behavior of HDPE-TiO_2_ nanocomposites were evaluated in a TA instrument (Model Q 200, TA Instruments, New Castle, DE, USA). Throughout the experiment, the system was maintained in a nitrogen environment to avert the oxidation of the sample; a heating rate of 10 °C·min^−1^ was used. Approximately, 5 mg of the sample was placed in a DSC pan and an empty pan were used as a reference. The DSC instrument was programmed to execute the analysis in three cycles. The first step involved a heating cycle in which the sample was exposed to a temperature from 25 °C to 200 °C. This was followed by a cooling cycle, which involved scanning the DSC pans with decreasing temperature from 200 °C to 25 °C. Finally, the sample was heated up to 200 °C to retrieve the melting DSC curve. After completion of each step, the system was maintained isothermally for 5 min to disregard any thermal history. The rate of crystallization was calculated from the formula given below [30]:

(1) $$\mathrm{Xc}=\frac{{\Delta H}_{m}}{\left(\Delta H^{{}^{\circ}}{˚}_{m}\right)\times \left(\varphi \right)}\times 100\%.$$

where, ΔH_m_ is the heat of fusion and ΔH°_m_ is as per literature the extrapolated value of enthalpy corresponding to 100% crystalline polyethylene; ΔH°_m_ = 293 J/g, φ weight fraction of HDPE [31].

#### 2.2.3. XRD Analysis

The crystal morphology of TiO_2_ loaded in HDPE was examined in an X-ray diffractometer (X’Pert^3^, PANalytical X-ray diffraction system, PANalytical, Denver, CO, USA). The X-ray diffraction pattern was obtained by using CuKα radiation (λ = 1.54 A°), operating at a voltage of 45 kV and under a current of 40 mA. To measure the distances between the atomic planes (i.e., d spacing) using Braggs law the following equation was used [32]:

(2) nλ=2dsinθ

Crystallite size of the nanocomposites were measured by using the Scherrer equation [33].

(3) $$L_{\mathrm{hkl}}=\frac{K\lambda }{\beta \cos\theta }$$

where λ (A°) is the wavelength of the X-ray, θ is the angle the between the incident X-ray and the plane perpendicular to the (hkl) plane, *K* is the crystallite shape factor which is 0.9 here, and β is the width of the diffraction beam at half height.

#### 2.2.4. Scanning Electron Microscopy (SEM)

The surface morphology of the nanocomposites was examined by means of SEM (FEI Quanta 50, Hillsboro, OR, USA) using a secondary electron detector (SE), at 30 kV and a working distance of 9.6 mm. Samples were sputter coated with a nano film of Au/Pd using a 108 Auto Sputter Coater (Cressington Scientific Instruments, Watford, UK). EDS (energy dispersive spectroscopy) mapping of the surfaces was supported by Oxford systems, Abingdon, UK.

#### 2.2.5. Fourier Transform Infrared (FTIR)

The presence of particular functional groups and bonding was analyzed by performing infrared spectroscopy using a Fourier transform spectrophotometer by SHIMADZU (Model: 8400S) in the range from 650 cm^−1^ to 4000 cm^−1^.

## 3. Results and Discussion

### 3.1. Surface Morphology and Nanoparticles Dispersion

**Figure 1:**
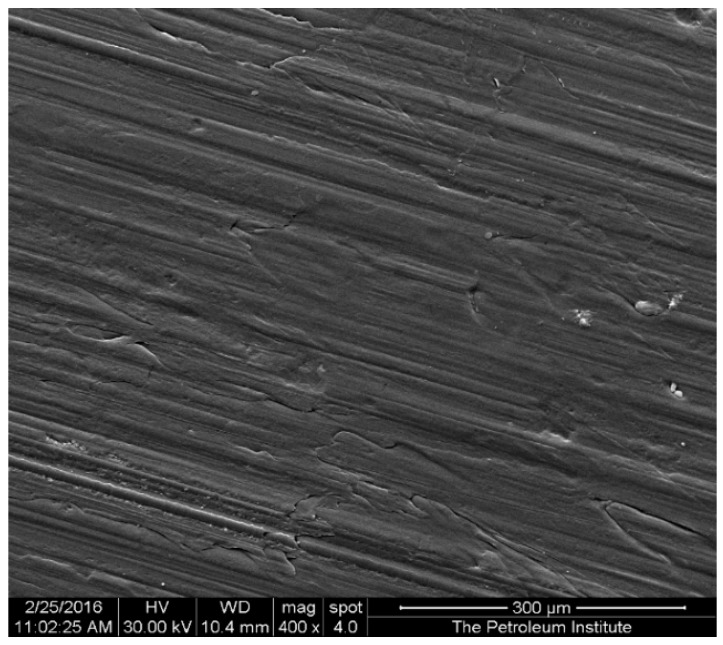
Representative secondary electron (SE) scanning electron microscopy (SEM) micrograph of high density polyethylene (HDPE)/TiO_2_ composites with 5 wt % nano-TiO_2_ prepared at a barrel temperature of 225 °C for 50 min of residence time. Image taken at 400× magnification and scaled to 300 micrometers.

In this study, TiO_2_ nanoparticles enriched HDPE nanocomposites were fabricated using injection molding under varying operating conditions. In order to analyze the nanocomposites’ surface topography and to verify the presence of titania particles, SEM images were taken for the developed HDPE-TiO_2_ nanocomposites. Figure 1 is a representative micrograph showing the surface morphologies of the composites that were prepared with a consistent TiO_2_ content of 5 wt % , barrel temperature of 225 °C, and a residence time of 50 min. Micrographs for different barrel temperatures (150 °C, 225 °C, 250 °C, and 300 °C) were obtained. The SEM micrographs show some irregularities on the surfaces of the injection molded nanocomposites samples and no evidence of agglomeration at the selected magnification were observed. According to Rideal et al. [34] temperatures for HDPE at or above 300 °C resulted in degradation of the polymer due to the thermal effects on the bonding of the structure. Moreover, practically at high barrel temperatures in the injection molding equipment with samples containing metal fillers, the samples tend to overheat resulting in a less desirable surface [35]. A similar result was also confirmed by Tripathi et al. [3] who reported a rougher surface when two or more additives were added to the polymer matrix when samples were prepared at 180 °C [3].

**Figure 2:**
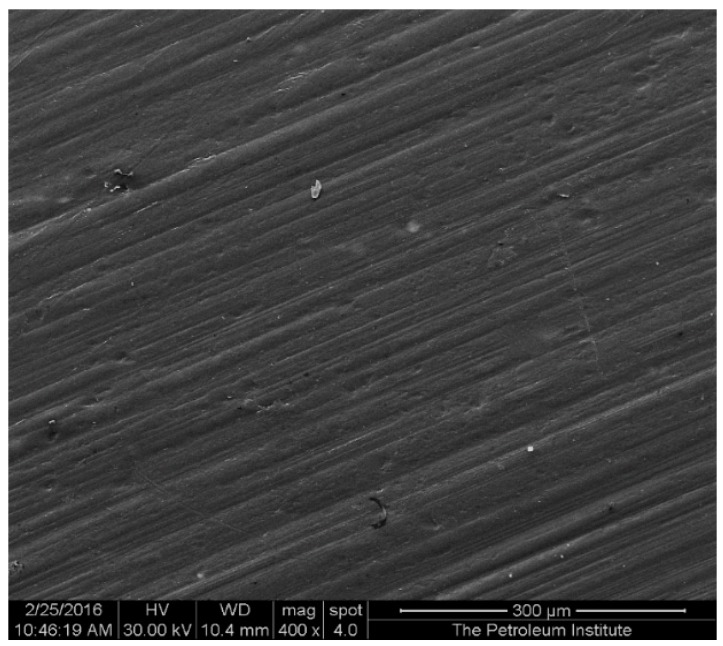
Representative secondary electron (SE) SEM micrograph of HDPE/TiO_2_ composites with 5 wt % nano-TiO_2_ prepared at a barrel temperature of 250 °C for 30 min residence time. Image taken at 400× magnification and scaled to 300 micrometers.

Nanocomposites consisting of 5 wt % TiO_2_ were developed at a fixed barrel temperature of 250 °C, with varying residence time of 30 min, 50 min, and 70 min. It was observed that residence time as an independent variable does not affect the surface morphology of the injection molded nanocomposites. Figure 2 is a representative micrograph prepared at 30 min residence time. The surface of the samples is dominated by apparent ridges. In agreement with the observations, Mozumder et al. [36] confirmed the presence of nano-topographies consisting of groves and numerous concavities all over the polymeric surface. Also, Shi et al. [37] examined polymeric surfaces by SEM and confirmed the presence of micro-sized titanium dioxide particles on a smooth polymeric coating; other studies by the group also supported these observations [38,39,40]. Ranjusha et al. [41] processed hybrid composites at two different barrel temperatures (180 °C and 190 °C) during injection molding, and their findings concluded that the temperature had little effect on the overall morphology of the composites [41].

**Figure 3:**
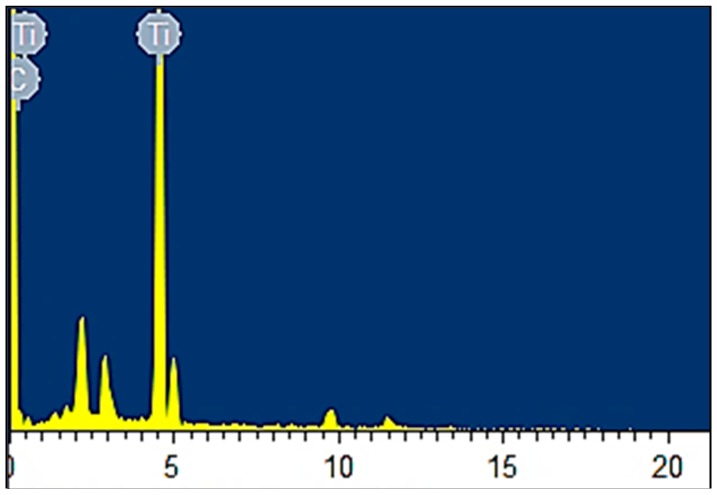
Elemental composition analyzed by energy-dispersive X-ray spectroscopy (EDS) on HDPE/TiO_2_ nanocomposite at 5 wt % injection molded under a barrel temperature of 250 °C and residence time of 50 min.

HDPE/TiO_2_ nanocomposites fabricated through injection molding with 5% titania were analyzed by energy-dispersive X-ray spectroscopy (EDS) to confirm the presence and even distribution of the metal oxide filler. Figure 3 shows the composition analysis of part of the surface region of the composite (fabricated at 250 °C) and it can be clearly inferred that the principal component (carbon) is found abundantly on the surface since HDPE is the composite matrix with the hydrocarbon chain; titanium is also present in high composition in the matrix. To further elucidate the finding above, elemental mapping, also by the means of EDS was conducted on the sample in order to show the distribution of the different chemical elements present in the composite.

**Figure 4:**
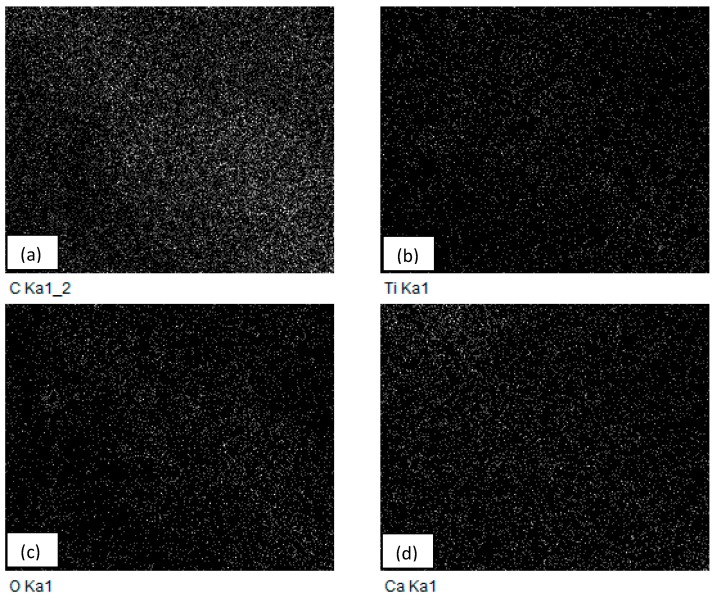
A representative elemental mapping by EDS spectroscopy on injection molded HDPE/TiO_2_ nanocomposite fabricated under a barrel temperature of 250 °C and a residence time of 50 min. Images represent the distribution of the different chemical elements on the nanocomposite surfaces: (**a**) carbon; (**b**) titanium; (**c**) oxygen; and (**d**) calcium.

Figure 4a–d shows that the carbon content is much higher than the other elements. Titanium elemental mapping resulted in a uniformly distributed image with titania particles dispersed all over the nanocomposite surface. Similar results were achieved by Mozumder et al. [42] in which the polymeric powder coatings (PPC) resulted in an even distribution of micrometer-sized titanium across the different surfaces. Also, Wang et al. [9] confirmed the presence of titanium through point EDS analysis, resulting in the successful incorporation of the filler. Other elements such as oxygen were also present since titanium dioxide was the filler component and the presence of calcium was observed in significant amounts as it is a common additive found in commercial grade metal oxides.

Therefore, elemental mapping confirms the presence of titania nanoparticles in the HDPE matrix with a good overall dispersion. Nanocomposite prepared at a barrel temperature of 150 °C exhibited a non-uniform morphology, while composites at higher temperatures consisted of regular ridges and pores. The surface morphologies of the nanocomposites with varying residence time showed little variation but remained consistent with the general findings.

Infrared (IR) analysis is a very useful technique when analyzing different compounds since the spectra result in molecular fingerprints that enable identification of various organic and inorganic molecules [28,43]. IR absorptions are useful in determining the presence of different bond stretching pertaining to a particular chemical bond, the percent transmittance, as well as the intensity of a particular peak [44].

**Figure 5:**
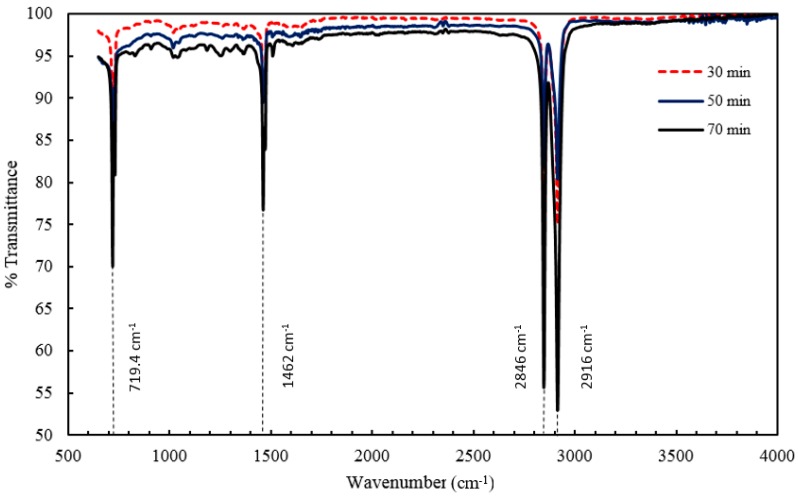
Fourier transform infrared (FTIR) spectra of injection molded HDPE/TiO_2_ nanocomposites at 5 wt % TiO_2_ and a barrel temperature of 250 °C. Residence time was varied at 30 min, 50 min, and 70 min.

**Figure 6:**
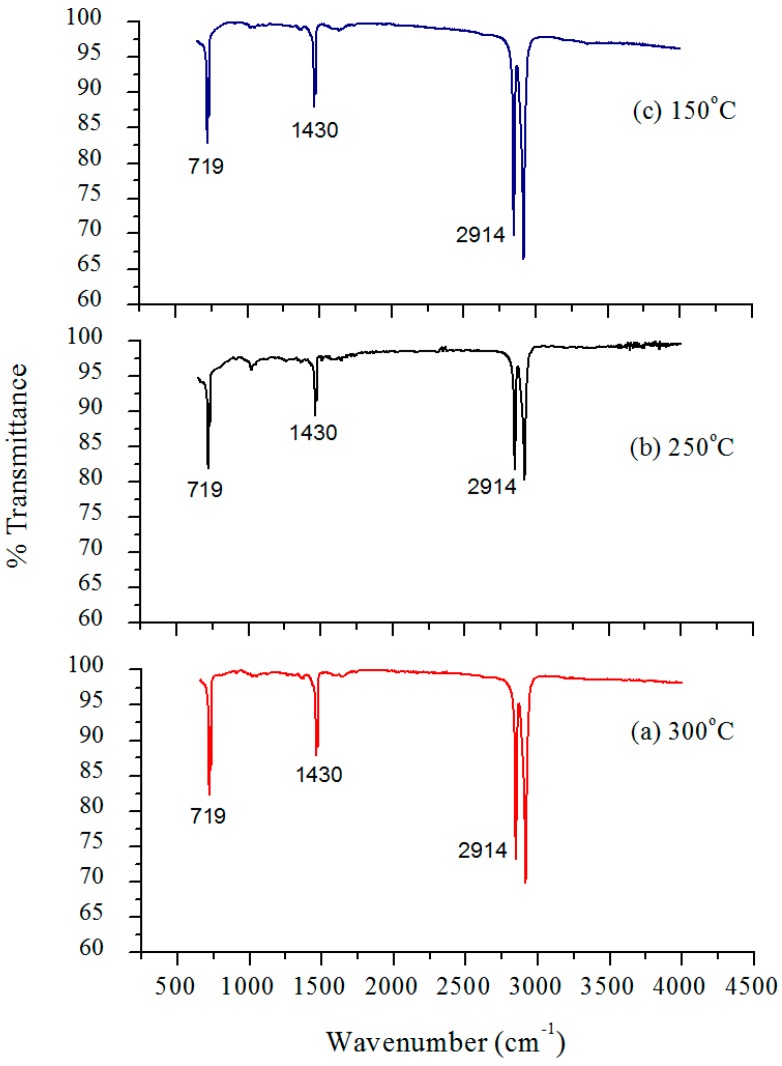
FTIR spectra of injection molded HDPE/TiO_2_ nanocomposites at 5 wt % TiO_2_ and residence time of 50 min. Barrel temperature was varied at: (**a**) 300 °C; (**b**) 250 °C; and (**c**) 150 °C.

FTIR spectra of all injection molded nanocomposites were recorded by the means of a reflective IR technique that absorbed radiation from the surface of the nanocomposites. Figure 5 and Figure 6 show the spectra of different samples with varying residence time and barrel temperature, respectively. HDPE being an organic compound resulted in characteristic organic peaks that were observed in the range 2850–2950 cm^−1^ [45], specifically representing the alkyl C–H bond seen in both Figure 5 and Figure 6.

The most significant peak was obtained at 1430 cm^−1^ that in turn confirmed the presence of titanium dioxide in the polymeric matrix and is characteristic of stretching and vibration of the Ti–O–Ti bond [19]. Vibrations in the range of 466–700 cm^−1^ are due to the single bond between the titanium and oxygen atom, Ti–O, and often present in the stretching of the titanium dioxide molecule [19]. The presence of titanium dioxide on the surfaces of all the nanocomposites was hence confirmed. FTIR can also be helpful in detecting any microstructural changes in a compound. Peaks at 1410 cm^−1^ and 1375 cm^−1^ occurred due to the symmetric bending of the –CH_3_ group and the scissoring of a long chain alkyl group, respectively [25]. Also, peak at 1462 cm^−1^ is attributed to the vibrational changes occurring in the C–H bond [46].

In reference to Figure 5, it can be inferred that, the considered residence time range has no effect on the overall infrared spectra of the nanocomposites, as all the characteristic peaks are well aligned with each other. In addition, the intensity varies in the lower range of the wavenumbers in which 30 min shows the highest transmittance followed by 50 min and 70 min with values of 85%, 82%, and 70% respectively. The decrease in transmittance could be explained by the occurrence of any bond weakening or cleavage when the melted composite mixture remains in the barrel for a prolonged period, nevertheless the spectrum converges along its length [47]. Figure 6 presents a stacked graph of the barrel temperature samples, and although similar to the trend found in the residence time, a wider range of transmittance is observed at 150 °C (between 68% and 97%).

### 3.2. Mechanical Characterization

**Figure 7:**
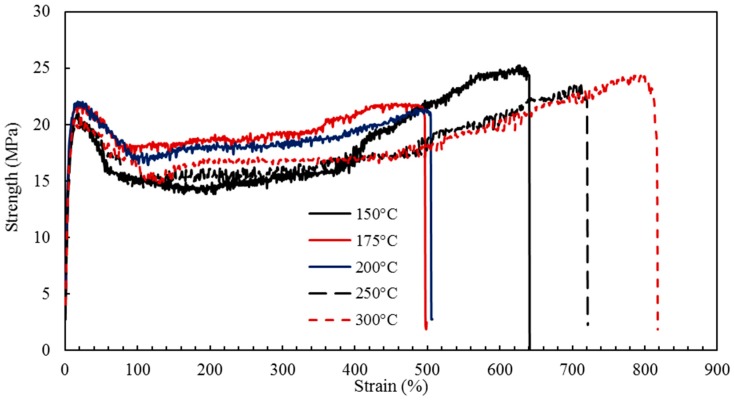
Stress-strain curves of injection molded nanocomposites with varying barrel temperature (BT), nanocomposites BT1–BT5 at 150 °C, 175 °C, 200 °C, 250 °C, and 300 °C, respectively.

In the selection of biomaterials based on their optimum functionality, mechanical characterization of the nanocomposites is a crucial analysis that serves to quantify some of the important mechanical characteristics including tensile and yield strengths, modulus of elasticity, and percent elongation. For the present work, in order to investigate the effects of barrel temperature on the mechanical properties of the HDPE-TiO_2_ nanocomposites, uniaxial tensile testing was conducted. The TiO_2_ content of 5 wt % and residence time of 50 min were fixed so that only the effect of barrel temperature (150, 175, 200, 250, and 300 °C) was studied. The tensile strength, elastic modulus and percent elongation were obtained from the stress-strain curves for all the nanocomposites and representative curves are presented in Figure 7. The current experimental data, along with some of the work from previous literature are reported in Table 1.

**Figure 8:**
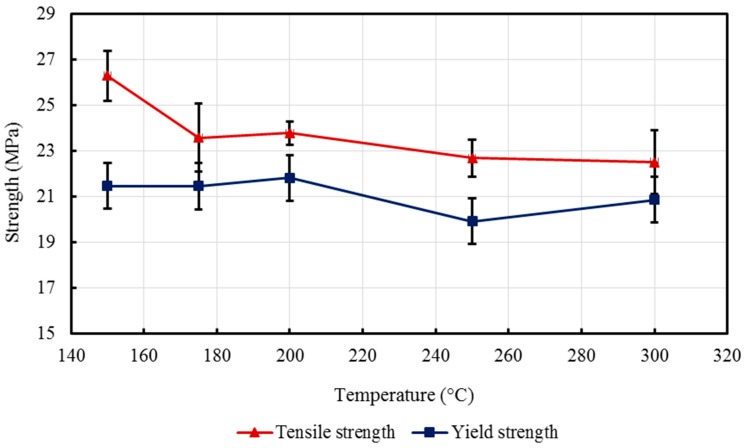
Effect of barrel temperature on tensile and yield strengths of the developed HDPE-TiO_2_ nanocomposites (SE bars included).

The yield strength of the nanocomposites, BT1–BT5, ranges from 19.94 MPa to 21.85 MPa. This narrow range illustrates that the yield strength is not significantly affected by the considered range of barrel temperatures. The tensile strength, or the fracture strength, for the nanocomposites was found to vary within the range from 22.53 to 26.30 MPa. Table 1 and Figure 8 show the change in the yield and tensile strengths with the barrel temperature, with standard error (SE) bars. It can be noted that at a barrel temperature of 150 °C, the composite revealed the highest tensile strength and lower values were found for higher barrel temperatures. This is possibly attributed to the phase size of the specimens that were injection molded at high temperatures [52]. A study by Zhou et al. [53] on examining the effect of melt temperature also supported this trend; talc-reinforced polypropylene injection molded composites revealed a lower yield strength at high melt temperatures (barrel temperature). The tensile strength nevertheless decreased due to the interfacial binding between the metallic/ceramic filler and polymer matrix [3].

**Figure 9:**
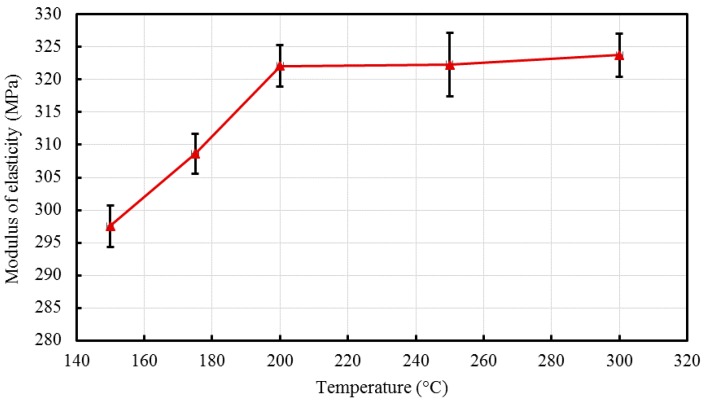
Effect of barrel temperature on Young’s modulus of the developed HDPE-TiO_2_ nanocomposites (SE bars included).

Young’s modulus, which represents the stiffness of the material was calculated from the initial linear region of the stress-strain curves, the average values of three samples were considered and are shown in Table 1 and presented in Figure 9, with SE bars. The moduli of the nanocomposites were found to have little variation and were observed to increase with barrel temperature; a total increase of 8.81% was shown when the barrel temperature increased from 150 °C to 300 °C. Tripathi et al. [3] also studied HDPE composites incorporated with alumina and hydroxyapatite that were fabricated using injection molding at 180 °C (see Table 1). The mechanical tests revealed that the minimum modulus of elasticity occurred with neat HDPE and consistently increased at higher filler concentrations.

**Figure 10:**
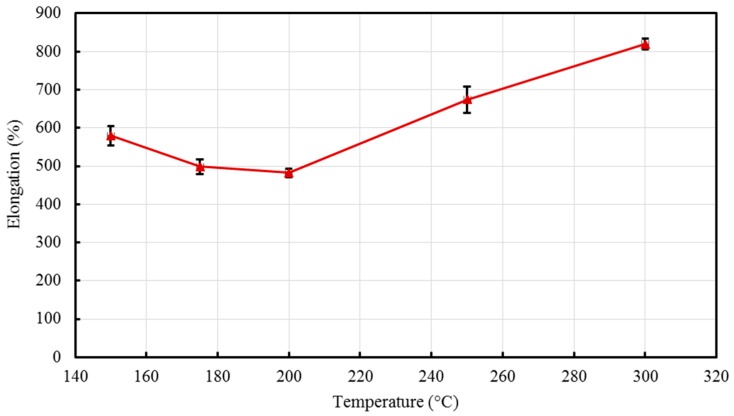
Effect of barrel temperature on percent elongation of the developed HDPE-TiO_2_ nanocomposites (SE bars included).

The percent elongations, representing the ductility of the material were obtained and are demonstrated in Figure 10. At 150 °C, the elongation of the composite was found to be ≈580%, while at 200 °C the elongation observed had a slightly lower value ≈482%. This could be explained by the presence of laminar orientation occurring at low temperatures that resulted in higher ductility, this result was also observed by Zhang et al. [52] in which PP/LLDPE composites revealed higher percent elongation at a low melt temperature (barrel temperature).

It is evident that HDPE/TiO_2_ nanocomposites processed at higher barrel temperatures showed improved ductility and an increase in the modulus of elasticity when compared to the lower barrel temperatures. In agreement with the aforementioned results, Khan et al. [5] modeled the feasibility of recycled HDPE compared to pure, together with the optimization of the injection molding operating parameters [5]. According to the grey relational analysis performed by their group, a barrel temperature of 240 °C was concluded to be the optimum for the processing of neat HDPE [5]; as the barrel temperature was increased from 200 °C to 240 °C, a slight increase in tensile strength was observed. Moreover, the effect of processing conditions was studied by Mourad et al. [54,55] on die drawn polypropylene, and it was found that the compression modulus and yield strength increased with a higher processing temperatures. Zhang et al. [52] studied the effect of processing temperature on PP/LLDPE injection molded bars and observed a trend similar to the present study, that is, at high temperatures, the strength of the polymeric materials was found to be low.

**Figure 11:**
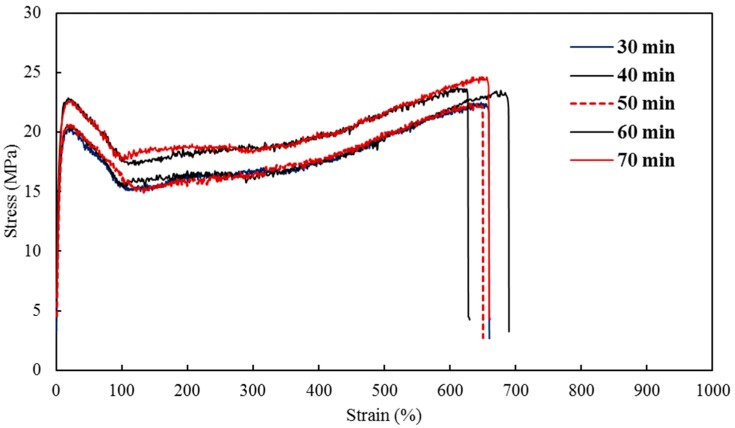
Stress-strain curves of injection molded nanocomposites with varying residence time.

To further study the operating conditions of injection molding, the residence time was varied in the preparation of the nanocomposites between 30 min and 70 min. A filler concentration of 5 wt % remained constant as well as the barrel temperature at 250 °C. The nanocomposites were subjected to tensile testing to evaluate the different parameters important for biomedical applications. The stress-strain curves for the nanocomposites (RT1-RT5) are shown in Figure 11.

**Figure 12:**
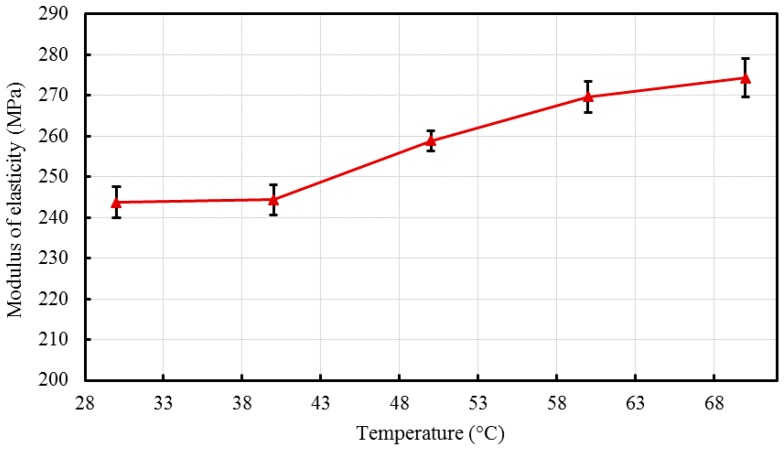
Effect of residence time on the modulus of elasticity of the developed HDPE-TiO_2_ nanocomposites (SE bars included).

In general, the residence time may not result in remarkable differences between the properties of the composites [14,56]; however, it is still imperative to consider its effect by varying it during the processing. The tensile strength and yield strength, along with the modulus and percent elongation are reported in Table 2. Young’s moduli were found to be 243.76 MPa, 244.38 MPa, 258.88 MPa, 269.67 MPa, and 274.34 MPa for nanocomposites RT1-RT5 respectively. A slow and consistent increasing trend was observed which is better represented in Figure 12 with SE bars.

**Figure 13:**
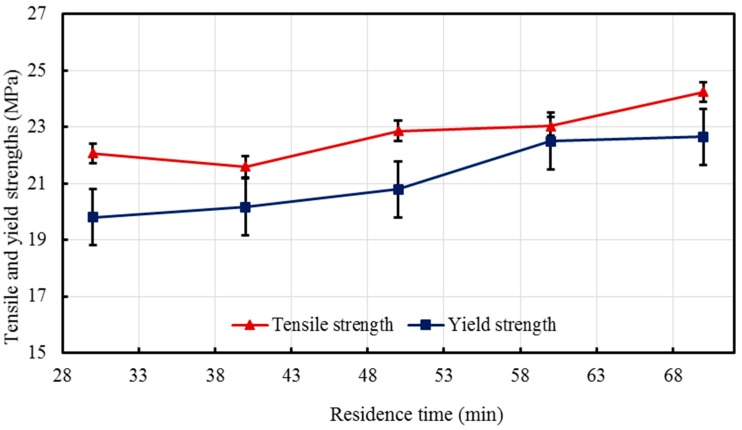
Effect of residence time on yield and tensile strengths of the developed HDPE-TiO_2_ nanocomposites (SE bars included).

Figure 13 demonstrates the variation of the yield and tensile strengths with barrel residence time. The same slow increasing trends analogous to that of the modulus of elasticity are observed. The yield strength of the composites varies with a maximum range of 2.85 MPa, showing negligible variation when the residence time was altered.

The tensile strength demonstrates the same trend modulus and yield strength, with a highest value of 24.25 MPa and a lowest value of 21.60 MPa.

**Figure 14:**
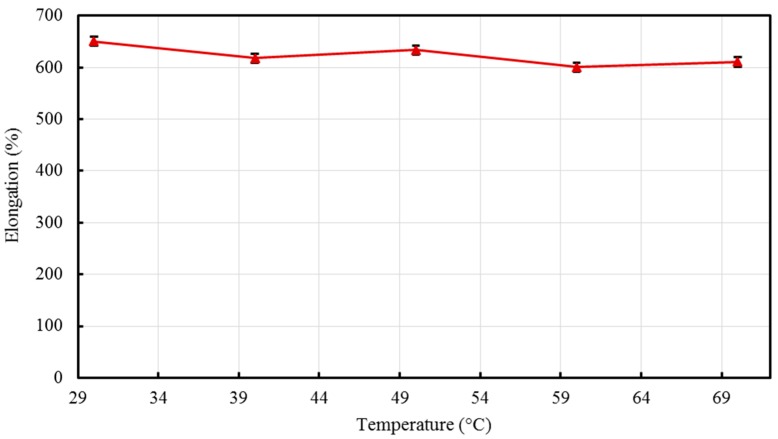
Effect of residence time on percentage of elongation of the developed HDPE-TiO_2_ nanocomposites (SE bars included).

The percent elongation values of the composites vary within a range of 49.4% (see Figure 14) with residence time, which is relatively a narrow range. In light of the above results, it can be concluded that the considered range of residence time (30 min to 70 min) has a minor effect on the mechanical performance of HDPE/TiO_2_ nanocomposites.

In a study performed by Bociaga and Palutkiewicz [26], the injection time (residence time) had negligible effect on the mechanical properties of injection molded HDPE parts. Zhil’tsova et al. [24] also performed a similar study to understand the effects of different processing conditions on injection molded HDPE acetabular cups. Their results revealed that when the injection time was reduced, the cups obtained were of lower weight [24].

### 3.3. Thermal Analyses 

#### 3.3.1. TGA (Thermogravimetric Analysis)

For large scale production of nanocomposites, thermal stability plays an important role in obtaining superior quality products. In this study, the thermal stability of nanocomposites was evaluated using TGA and DSC techniques. In TGA, physical and chemical changes upon heating at constant rate are noted as a function of temperature and time. The degradation temperature and highest degradation rate-temperature of the fabricated nanocomposites are obtained from the respective TGA thermograms [57].

**Figure 15:**
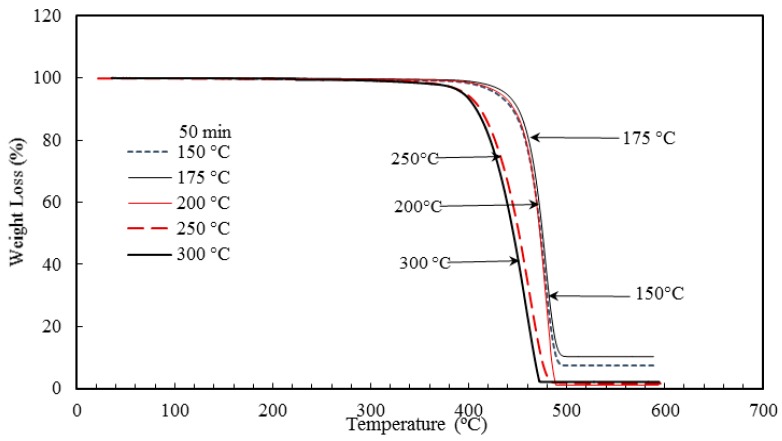
Thermogravimetric analysis (TGA) thermograms of HDPE/TiO_2_ nanocomposites with varying barrel temperature.

**Figure 16:**
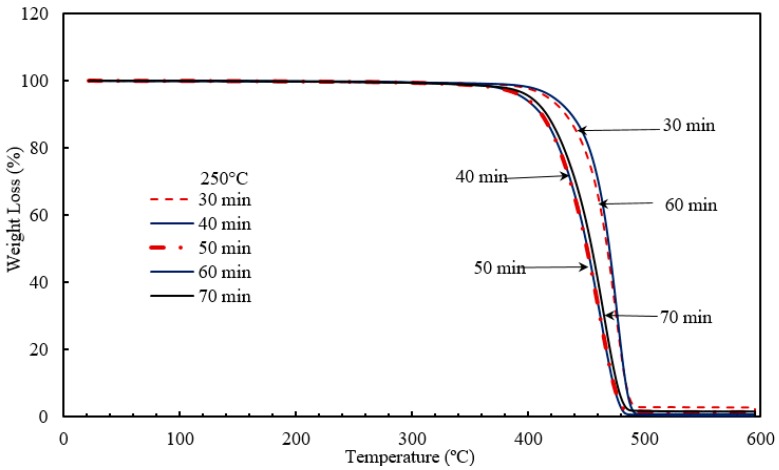
Pictorial representation of TGA analysis carried out on HDPE-TiO_2_ with varying residence time.

The thermograms of the manufactured nanocomposites (BT1-BT5 and RT1-RT5) were taken from thermogravimetric analysis (TGA). Nanocomposites containing 5 wt % TiO_2_ nanoparticle were injection molded with a different set of barrel temperatures and residence times. The thermograms were obtained by degrading the composites until complete degradation was witnessed As shown in Figure 15 and Figure 16, it can be seen that temperature and residence time affect the TGA. According to Beyler and Bayar et al. [58,59] the stiffer the polymer, the greater is its melting temperature. However, at higher temperatures the polymer tends to become ductile in nature and the melting temperature starts decreasing and the polymer starts degrading. When a temperature exceeds its withstand point, polymer fragmentation occurs and the polymer decomposes. Heating the polymer below its temperature of degradation and cooling the polymer helps to bind it with the nanoparticles which results in a composite more stiffer in nature. With the escalation of the temperature, polymeric materials lose their stiffness and become more ductile in nature, which in turn affects the thermal stability of the composites. The polymer, when exposed to certain temperatures, undergoes degradation and the interaction between the polymer matrix and nanofiller is changed. On annealing, superior quality nanocomposites are developed, even though a higher degree of the processing temperature has a negative impact on the thermal stability [60]. Table 3 and Table 4 summarize the results of TGA. As shown in Table 3, the rate of degradation of nanocomposites decreased as the sample was processed through high barrel temperatures. On the contrary, in the case of residence time, the higher duration gave better TGA results. However, with increase in barrel temperature and residence time, the maximum degradation temperature decreased.

Ranjusha et al. [41] investigated the influence of molding temperature on the injected molded composites and reported that with increase in temperature from 180 to 200 °C, decrease in thermal stability was observed. In another research, Boey et al. [58] found that at lower temperature and shorter residence time, complete melting of polymer and blending of fillers may not be achieved. On the contrary, Davis et al. [61] established that melt blending of poly(ethylene terephthalate) clay nanocomposites for longer duration and higher screw speed resulted in low quality products.

To conclude, the highest rate degradation temperature of nanocomposites was 459.5 **°**C, which was obtained at 175 **°**C temperature and 50 min residence time. As mentioned above, initially the polymers, under the influence of temperature, melt and a certain amount of degradation takes place. On annealing, polymer will bind with nanoparticles which in turn results in better thermal stability. However, when processing conditions exceed the limit, polymer degradation becomes irreversible and significant changes occur in the structure of the material. The polymer loses its properties and leads to fragmentation [60,62]. In this study, it was noticed that the degradation temperature approximately reduced by 34.5 **°**C with the increase of barrel temperature from 150 to 300 **°**C. Similarly, in case of residence time, with escalation of time from 30 to 70 min, there was a decrease of 19 **°**C in degradation temperature. This decrease in thermal stability can be attributed to the fact that as the duration increases. the polymer has a longer time to melt and degrade. This irreversible degradation can be one of the reasons for the decrease in thermal stability with increase in residence time [60,63].

#### 3.3.2. DSC (Differential Scanning Calorimetry)

In designing the polymeric nanocomposites, tuning the operating conditions becomes extremely crucial to ensure efficient dispersion of nanofillers in the polymeric matrix. For example, each polymer has its own degradation temperature and while choosing the operating conditions, it is very important to choose a temperature lower than its degradation temperature and follow a method that enhances the thermal stability of the nanocomposites [64]. Hence, differential scanning calorimetry was used to obtain the melting and crystallization temperature of nanocomposites along with the degree of crystallinity.

**Figure 17:**
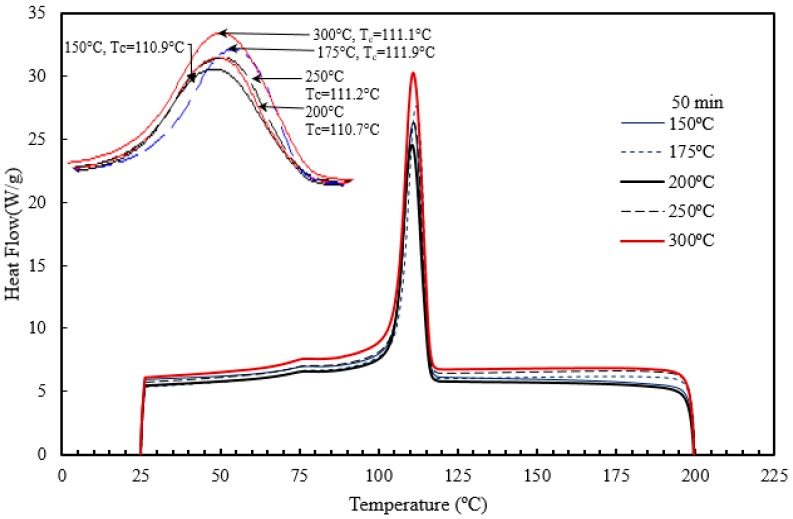
Effect of barrel temperature on the crystallization temperature of the injection molded HDPE/TiO_2_ nanocomposites.

**Figure 18:**
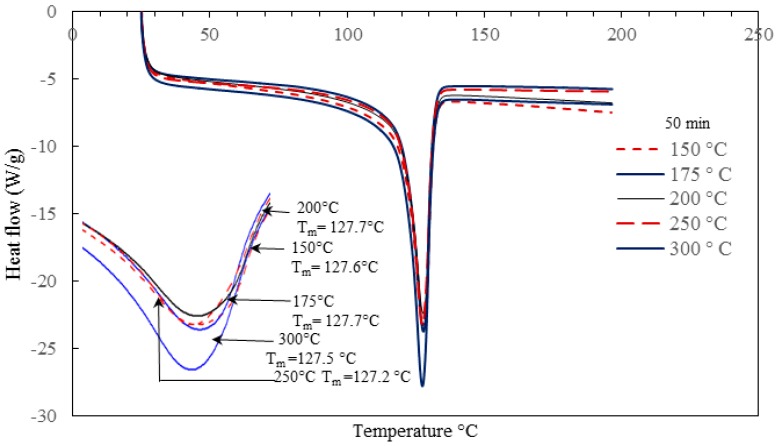
Effect of barrel temperature on the melting temperature of the injection molded HDPE/TiO_2_ nanocomposites.

In the current work, the DSC curves for polymeric nanocomposites were generated for different barrel temperatures (BT1–BT5) and residence time (RT1–RT5) under a nitrogen atmosphere. Figure 17 and Figure 18 are the pictorial representation of the DSC melting and cooling curves generated under different barrel temperatures. Table 5 summarizes the values of some of the kinetic parameters (e.g., the melting and crystallization temperatures and degree of crystallinity). The results reveal that, the melting and cooling curves of the nanocomposites have not been affected significantly by the range of barrel temperatures studied. Increasing the barrel temperature from 150 °C to 300 °C leads to a general slow increasing trend in the rate of crystallization (from 60.7% to 74.1%). Similar results were reported by Hedesiu et al. [65], on studying the effect of temperature on high-density polyethylene. They postulated that, with an increase in temperature, the molecular movement of polymers began to shift from amorphous to lamellae surface. On annealing, there is a shift in the reorganization of the amorphous and the crystallization region, this in turn, boosts the arrangement of the crystallization order. Due to regular shifts in the amorphous region, the thickness of the amorphous area reduced, as a result of which more crystals were exposed [65].

**Figure 19:**
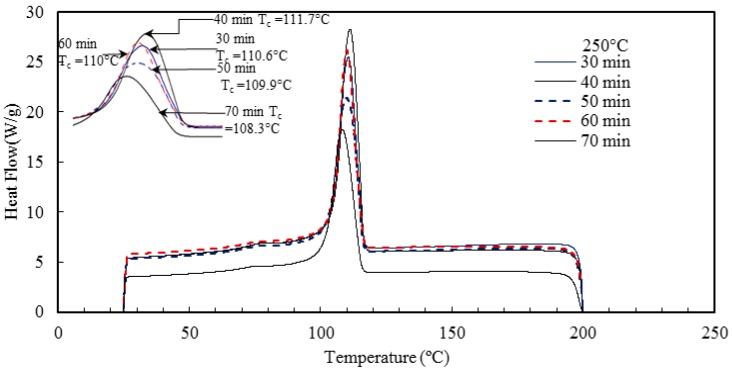
Effect of barrel temperature on the crystallization temperature of the injection molded HDPE/TiO_2_ nanocomposites.

**Figure 20:**
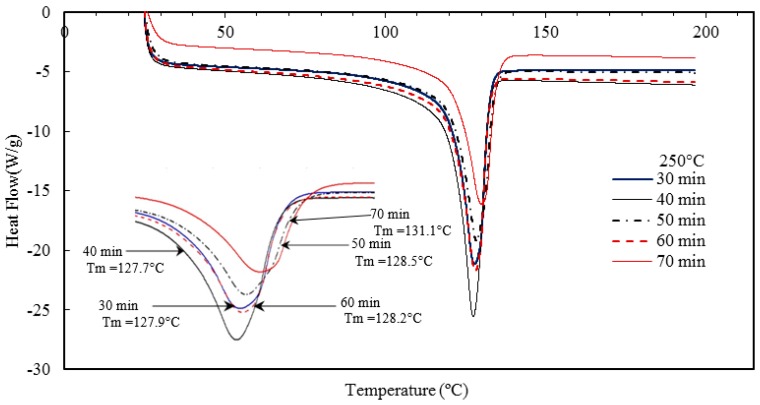
Effect of residence time on the melting temperature of the injection molded nanocomposites.

The temperature at which the last trace of crystal vanishes is defined as the melting temperature. Figure 17 and Figure 18, and Table 5 indicate that the barrel temperature has a minor effect on the melting and crystallization temperatures. Similar research was conducted by Ranjusha et al. [41] who examined the influence of barrel temperature on the hybrid nanocomposites. They summarized that for injection molded nanocomposites, the barrel temperature has a significant effect on the thermal and mechanical stability of the samples. Bociaga et al. [26] analyzed the influences of injection temperature, injection velocity, and mold temperature on injection molded high-density polyethylene. They concluded that the injection temperature and injection velocity have a slight influence on the improvement of the crystallinity of the nanocomposites. However, the increment of the mold temperature increased the degree of crystallinity. They suggested that with an increase in mold temperature, the polymer of interest has a longer time to cool down in the mold cavity and at high temperature, the polymer tends to form larger pores. As a result of this, the product has a higher degree of crystallinity [26]. The effect of residence time on the melting and crystallization temperature, and degree of crystallization is presented in Figure 19 and Figure 20 and Table 6.

Influence of TiO_2_ on the crystal morphology of HDPE was also evaluated using X-ray powder diffraction (XRD). It is a powerful technique in determining the crystal’s interplanar distances, size and its structure [18,66].

**Figure 21:**
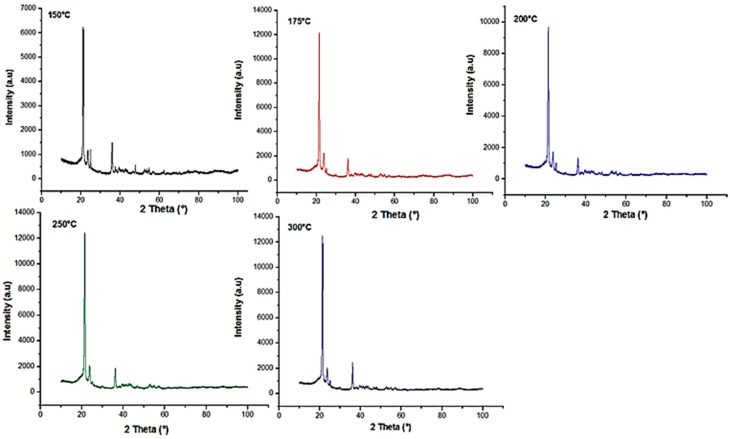
X-ray diffraction pattern for HDPE/TiO_2_ nanocomposites fabricated at various barrel temperatures.

**Figure 22:**
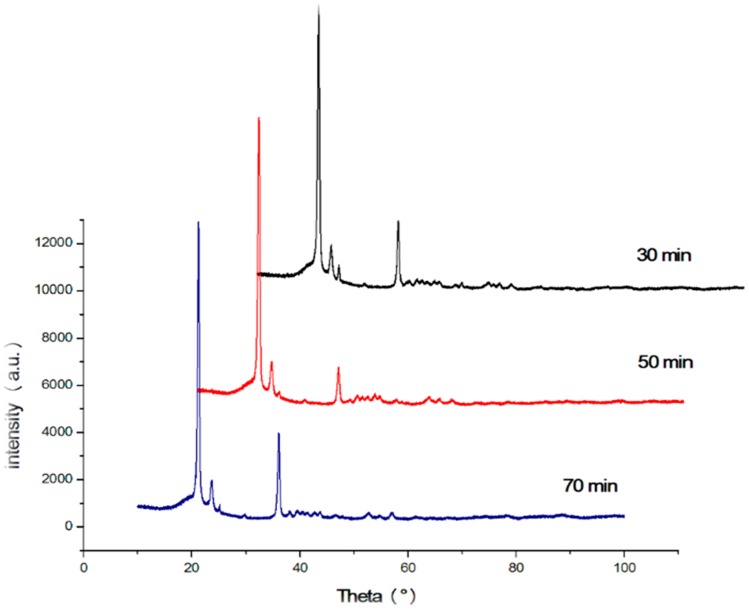
X-ray diffraction pattern for HDPE/TiO_2_ nanocomposites fabricated at various residence times.

The standard diffraction peak for high-density polyethylene is situated at 2θ = 21.4° and 23.7° which are in agreement with the diffraction peaks obtained [67,68]. The presence of TiO_2_ in the nanocomposites was detected using XRD patterns. From the literature, it can be concluded that XRD patterns of TiO_2_ shows peaks at 25.4°, 37.9°, and 48.1° [8,69]. As seen from Figure 21 and Figure 22, the diffraction pattern shows prominent peaks at 21.4°, 23.7°, and 36.1° implying that TiO_2_ is evenly distributed in the polymeric matrix. Table 7 and Table 8 show a summary of interplanar distances and crystal sizes (L_hkl_) calculated from the diffraction peaks. However, from Table 7 and Table 8 it can be inferred that the interplanar distances (i.e., d-spacing) are not significantly affected by barrel temperature or residence time.

According to Wang et al. [70], the addition of TiO_2_ onto HDPE matrix has no effect on the lattice parameter. However, these nanoparticles help to increase the laminar thickness which results in the formation of perfect crystals. The crystal size was evaluated using the Scherrer derived relationship [33]. Crystal size of the nanocomposites increased with the escalation of the residence time of the polymeric mixture in the barrel (Table 7). However, the rise in barrel temperature had an adverse effect on the crystal size (L_hkl_) (Table 8). Decrease in crystal size can be postulated in such way that with higher temperature and shorter residence time, the formation of cross-link bond, reorganization, and chain folding between polymers during the crystallization process is hindered, resulting in a distorted lattice and incomplete crystals [70].

From XRD patterns, it can be concluded that the nanocomposites had an even dispersion of nanoparticles in the polymeric matrix irrespective of barrel temperature and retention time. For crystal plane 110, with increase in barrel temperature from 150 °C to 300 °C the crystallite size decreased from 35.2 to 34.6, in turn indicating the formation of perfect crystals.

## 4. Conclusions

Nano-sized titanium dioxide enriched HDPE nanocomposites were fabricated through injection molding using dumbbell shaped inserts. In the injection molding process, the two main control variables are the barrel temperature and the residence time. Hence this study aimed to compare the nanocomposites’ mechanical, thermal, and structural properties obtained by changing these operating parameters. The SEM-EDS, FTIR, and XRD results confirmed the uniform distribution of TiO_2_ nanoparticles on the surface of the matrix. The degradation temperature decreased with an increase in the barrel temperature and residence time, however, the rate of crystallization showed a consistent rise in both cases; rate of crystallization was increased up to 75%.

The results revealed that there is no specific barrel temperature or residence time as an optimum value; for such nanomaterials, a range of temperature (150 °C–300 °C) can be considered for manufacturing these nanocomposites. However, the authors recommend researchers to use an operating barrel temperature lower than 300 °C since the polymer’s initial degradation temperature is about 424–460 °C. When samples were processed at temperatures around 300 °C, black spots were observed on the injection molded nanocomposites. Lastly, it is also recommended to use a temperature higher than 150 °C for easy material flow while injection molding. To conclude, the effect of varying the injection molding process parameters was studied and a wide range of barrel temperature and residence times can be used depending on the specific needs of the application.

## Figures and Tables

**Table 1:** Mechanical properties of injection molded high density polyethylene (HDPE) based nanocomposites from the current literature.

Current work & Literatures	Temperature (°C)	σ_y_ (MPa)	σ_u_ (MPa)	Modulus (MPa)	Elongation (%)
Current work 5% TiO_2_	150	21.5	26.3	297.5	579.8
	175	21.5	23.6	308.6	498.6
	200	21.9	23.8	322.1	481.9
	250	19.9	22.7	322.3	673.9
	300	20.9	22.5	323.8	819.4
Sotomayor et al. [48]	160	23.6	-	710.0	6.0
Kumar et al. [49]	165	-	19.9	592.0	10.2
Pegoretti et al. [50]	180	27.7	-	932.0	1173
Tripathi et al. [3]	180	-	24.2	564.3	855.4
Zhil’tsova et al. [24]	190–220	28.0	25.0	1300.0	-
Bartczak et al. [51]	190–200	24.9	14.5	756.1	730

**Table 2:** Summary of the results obtained from the mechanical tests performed on the injection-molded nanocomposites with varying residence times.

Sample	Designation	Residence Time (min)	Yield Strength (MPa)	Tensile Strength (MPa)	Young’s Modulus (MPa)	% Elongation
1	RT1	30	19.8	22.1	247.6	650.4
2	RT2	40	20.2	21.6	248.1	618.2
3	RT3	50	20.8	22.9	256.4	634.2
4	RT4	60	22.5	23.0	265.9	601.0
5	RT5	70	22.7	24.3	269.6	610.7

**Table 3:** Summary of thermogravimetric analysis (TGA) results showing the effect of barrel temperature on the degradation and the highest rate degradation temperatures of the fabricated nanocomposites.

Sample	Designation	Barrel Temperature (°C)	Degradation Temperature (T_d_) (°C)	Highest Degradation Temperature (T_dh_) (°C)
1	BT1	150	458.0	488.3
2	BT2	175	459.5	488.7
3	BT3	200	433.1	481.2
4	BT4	250	424.6	478.4
5	BT5	300	423.5	478.4

**Table 4:** Summary of TGA results showing the effect of barrel temperature on the degradation and the highest rate degradation temperatures of the fabricated nanocomposites.

Sample	Designation	Residence Time (min)	Degradation Temperature (T_d_) (°C)	Highest Rate Degradation Temperature (T_dh_) (°C)
1	RT1	30	450.4	485.8
2	RT2	40	426.8	480.7
3	RT3	50	424.5	478.4
4	RT4	60	454.9	486.9
5	RT5	70	431.1	479.5

**Table 5:** Differential scanning calorimetry (DSC) results showing the influence of barrel temperature.

Sample	Designation	Barrel Temperature (°C)	Degree of Crystallinity (%)
1	BT1	150	60.7
2	BT2	175	60.7
3	BT3	200	62.5
4	BT4	250	66.5
5	BT5	300	74.1

**Table 6:** DSC results showing the influence of residence time.

Sample	Designation	Holding Time (min)	Degree of Crystallinity (%)
1	RT1	30	67.9
2	RT2	40	75.8
3	RT3	50	66.4
4	RT4	60	58.2
5	RT5	70	56.4

**Table 7:** Summary of the parameters retrieved from the X-ray diffraction (XRD) curves.

Sample	Designation	Barrel Temperature (°C)	Crystal Planes	2θ (°)	d (A)	β (°)	L_hkl_ (nm)
1	BP1	150	110	21.2	4.2	0.40	35.2
			200	23.6	3.6	0.41	34.5
2	BP2	175	110	21.4	4.1	0.40	35.3
			200	23.8	3.7	0.47	30.2
3	BP3	200	110	21.4	4.1	0.43	34.6
			200	23.7	3.8	0.63	22.5
4	BP4	250	110	21.4	4.2	0.42	33.4
			200	23.7	3.7	0.61	23.2
5	BP5	300	110	21.4	4.2	0.40	34.6
			200	23.7	3.8	0.72	19.7

**Table 8:** Summary of the parameters retrieved from the XRD curves.

Sample	Designation	Residence Time (min)	Crystal Planes	2θ (°)	d (A)	β (°)	L_hkl_ (nm)
1	HT1	30	110	21.4	4.1	0.43	32.8
			200	23.8	3.7	0.52	27.3
2	HT3	50	110	21.4	4.2	0.42	33.6
			200	23.7	3.7	0.61	23.2
3	HT5	70	110	21.3	4.2	0.39	36.2
			200	23.6	3.8	0.55	25.8
